# Computational Approach Towards Exploring Potential Anti-Chikungunya Activity of Selected Flavonoids

**DOI:** 10.1038/srep24027

**Published:** 2016-04-13

**Authors:** Seyedeh Somayeh Seyedi, Munirah Shukri, Pouya Hassandarvish, Adrian Oo, Shankar Esaki Muthu, Sazaly Abubakar, Keivan Zandi

**Affiliations:** 1 Tropical Infectious Diseases Research and Education Center, Department of Medical Microbiology, Faculty of Medicine, University of Malaya, 50603 Kuala Lumpur, Malaysia

## Abstract

Chikungunya virus (CHIKV) is a mosquito-borne alphavirus that causes chikungunya infection in humans. Despite the widespread distribution of CHIKV, no antiviral medication or vaccine is available against this virus. Therefore, it is crucial to find an effective compound to combat CHIKV. We aimed to predict the possible interactions between non-structural protein 3 (nsP) of CHIKV as one of the most important viral elements in CHIKV intracellular replication and 3 potential flavonoids using a computational approach. The 3-dimensional structure of nsP3 was retrieved from the Protein Data Bank, prepared and, using AutoDock Vina, docked with baicalin, naringenin and quercetagetin as ligands. The first-rated ligand with the strongest binding affinity towards the targeted protein was determined based on the minimum binding energy. Further analysis was conducted to identify both the active site of the protein that reacts with the tested ligands and all of the existing intermolecular bonds. Compared to the other ligands, baicalin was identified as the most potential inhibitor of viral activity by showing the best binding affinity (−9.8 kcal/mol). Baicalin can be considered a good candidate for further evaluation as a potentially efficient antiviral against CHIKV.

Chikungunya virus (CHIKV) is an alphavirus transmitted mainly by female *Aedes aegypti* and *A. albopictus* mosquitoes. CHIKV was first isolated in Tanzania in 1952, and since the outbreak on Réunion Island in the Indian Ocean in 2005–2006, it has spread geographically over a vast region spanning more than 40 countries, including the United States and several European and Asian countries^1^,^2^. CHIKV is an enveloped virus with a single-stranded, positive-sense RNA as its genome of 11.8 kb in length. The genomic RNA consists of two open reading frames (ORFs), a 5′ end ORF that encodes nsP1-nsP4 and a 3′ end ORF that encodes the structural proteins including capsid, two major envelope (E) glycoproteins, E1 and E2 and two smaller accessory peptides, E3 and 6K^1^. nsP1 and nsP2 catalyse the synthesis of a negative strand of RNA with RNA capping properties. nsP2 also shows RNA helicase, phosphatase and proteinase activities. nsP3 has replication activity, whereas nsP4 has polymerase activity. In comparison with other CHIKV proteins, the function of alphavirus replicase protein (nsP3) is still uncertain, and there is presently no discovered inhibitor against this protein^3^. Normally, the onset of chikungunya symptoms begins between 4 to 7 days after a mosquito bite and is characterised by the abrupt onset of nausea, fever, headache, vomiting, fatigue, rash, myalgia and polyarthralgia^4^. Despite the importance of CHIKV infection for human health, there is presently no effective antiviral drug or vaccine available against CHIKV.

Plant-derived flavonoids are polyphenolic compounds endowed with a wide range of biological benefits to human health that include not only anti-inflammatory^5^, antioxidant^6^, antibacterial and antifungal activities^7^,^8^ but also antiviral activity^9^. The increase in the number of drug-resistant microorganisms has brought natural compounds such as flavonoids to the forefront as an important natural resource to overcome this problem. Large studies have successfully shown various types of flavonoids such as rutin, naringin, baicalein, quercetin and kaempferol^10^ to be potential antiviral agents against a wide range of important viruses including dengue virus^11^, HIV^12^, H5N1 influenza A viruses^13^, Coxsackie virus^14^ and Japanese encephalitis virus^15^. Effective flavonoids are also reported against CHIKV, including silymarin^16^ and luteolin^17^. However, none of these compounds are presently approved for the treatment of CHIKV infection^4^. Nevertheless, compounds such as ribavirin and chloroquine have been tested in clinical trials^1^,^18^.

The conventional method of performing *in vitro* cell culture-based assays previously used by researchers to discover new lead compounds with antiviral activity is costly for many investigators, especially if several compounds will be screened. To fill this gap in the field, instead of using the costly conventional method, we can use bioinformatics tools as a cost-effective solution for primary virtual screening of potential compounds.

Molecular docking, a branch of bioinformatics that accelerates the drug design process, is used in the biopharmaceutical industry to discover and develop new lead compounds^19^. The purpose of this assay is to predict a valid pose from a receptor conformation and a set of ligand conformations using scoring based on their binding affinity^20^. We recently began a series of *in vitro* studies on different potential flavonoids against CHIKV and have preliminarily observed *in vitro* anti-CHIKV activity of three selected flavonoids, namely baicalin, naringenin and quercetagetin, that is still being investigated (unpublished data). Therefore, we have designed the present study using computational approaches to discover the potential of these three flavonoids targeting nsP3 as one of the most essential viral elements for CHIKV intracellular replication.

## Results

**Figure 1:**
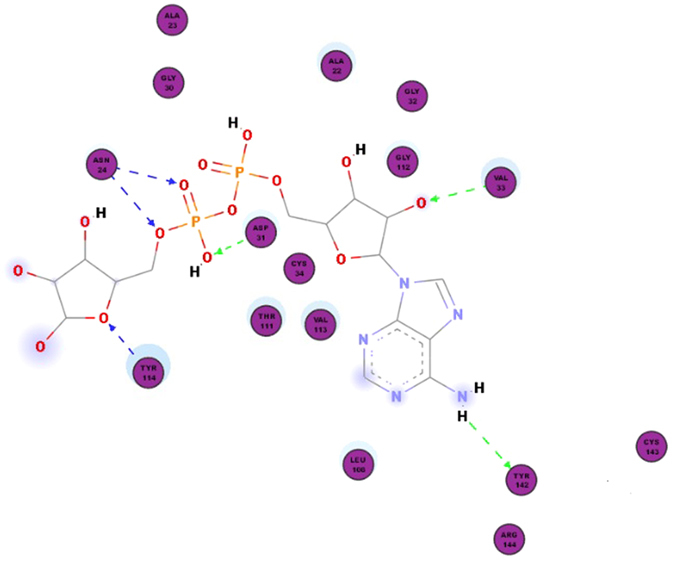
Re-docking ADP-ribose into ADP-binding site of nsP3. Close contacting residues of nsP3 (purple circles) when ADP-ribose was docked into the protein.

**Figure 2:**
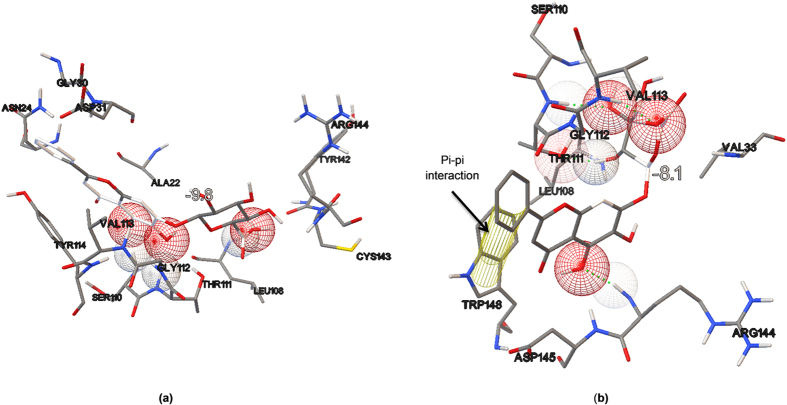
Intermolecular bonding between baicalin and nsP3. (**a**) Hydrogen bonding interaction between baicalin and nsP3 residue with binding affinity of −9.8 kcal/mol, which is the best compared to other ligands. (**b**) Pi-Pi interaction between baicalin with nsP3 residue, TYR114.

**Figure 3:**
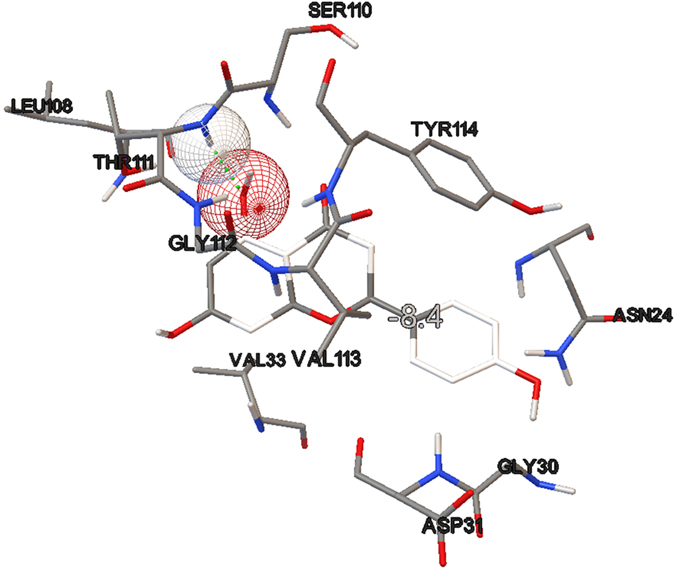
Hydrogen bonding interaction between naringenin and nsP3. The binding affinity obtained was −8.4 kcal/mol.

**Figure 4:**
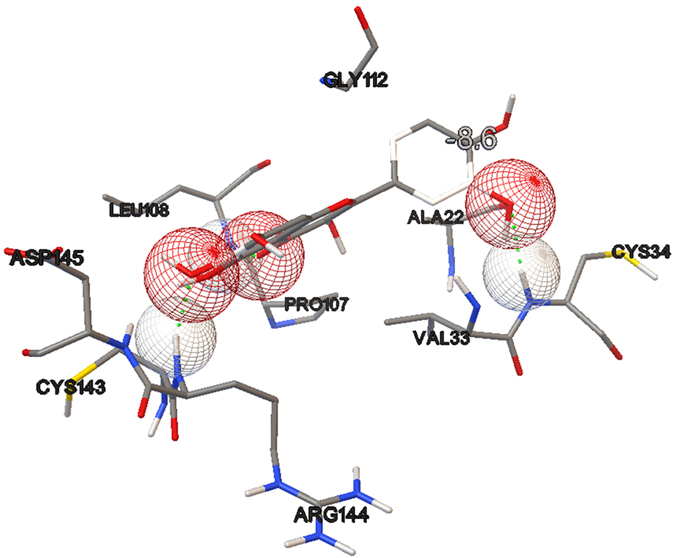
Hydrogen bonding interaction between quercetagetin and nsP3. Binding affinity of −8.6 kcal/mol was obtained.

The ligand conformations were ranked according to their predicted binding affinities using the default scoring function in AutoDock Vina. The nsP3 residues that formed close contacts with ADP-ribose are listed in Table 1 and shown in Fig. 1. The best docking conformation of ADP-ribose showed a binding affinity of −8.7 kcal mol^−1^, whereas among the three other ligands tested, baicalin showed the most potent antiviral activity with a binding affinity of −9.8 kcal mol^−1^. The binding affinities and their interaction energy are tabulated in Table 2. The intermolecular hydrogen bonds formed between each compound and nsP3, together with their distances, are presented in Table 3, which shows that the majority of the hydrogen-bond donors came from the protein residues and that the corresponding acceptors were derived from the ligands. It is evident that there were interactions of the ligands with 10 residues in the active site of nsP3 (baicalin: LEU108, TYR142, SER110, THR111; naringenin: SER110, THR111; quercetagetin: CYS34, LEU108, ARG144, ASP145). There was also one pi-pi interaction between baicalin with nsP3 residue TYR114 (Table 4). The best docking pose for each ligand was also recorded (Figs 2, 3, 4).

## Discussion

To date, only a little information is available on CHIKV nsP3 and its potential inhibitors. According to Rashad *et al.*^21^, the most promising targets from a chemical and biological standpoint are CHIKV nsP2 and E protein. Only one molecular docking study targeting nsP3 can be found in the literature; it reports on the combination of molecular docking, virtual screening and molecular dynamics simulations to identify the potential inhibitors of CHIKV nsP3^22^. Other molecular docking studies targeted CHIKV nsP2^23^, nsP4^24^,^25^ and viral E2 protein^26^. Considering the role of nsP3 in viral replication, the discovery of an nsP3 inhibitor is a major advance towards finding an active compound that can interfere with intracellular CHIKV replication. In the present study, we performed molecular docking using AutoDock Vina to study the antiviral activity of baicalin, naringenin and quercetagetin. The inhibitory effects of these three flavonoids against CHIKV *in vitro* replication were revealed during our other ongoing experimental studies (unpublished data), but their mechanisms of action remain unknown. Thus, the present study was conducted to evaluate the potential mechanism of action of these flavonoids based on the hypothesis that flavonoids can interfere with the viral replication cycle.

During the initial stage of the present study, ADP-ribose was re-docked into the nsP3 protein and managed to reproduce the important closely contacting residues in the ADP-ribose binding site in reference to the complex crystal structure from the Protein Data Bank (PDB ID: 3GPO). The results of the re-docking procedure comprising the closely interacting residues were used to evaluate the potential pharmacokinetic properties resulting from the binding sites of the subsequently tested ligands. Of the three tested ligands, baicalin showed the strongest interaction with nsP3 and showed the most potent potential anti-CHIKV activity, with a binding affinity of −9.8 kcal/mol and low Ki value of 0.064 μM, followed by quercetagetin and naringenin, with respective binding affinities of −8.6 and −8.4 kcal/mol. The Ki value or inhibitor constant is an indicator of the potency of an inhibitor, with a highly potent inhibitor being indicated by a low Ki value. Drugs with a Ki value <1 mM are normally considered to be effective^27^. Baicalin showed interaction with four residues of nsP3, LEU108, TYR142, SER110 and THR111, with distance ranges from 1.81482 to 2.42882. The most stable H-bond is that with an approximately 180° angle, i.e. close to linear^28^. A review by Szatylowicz^29^ classified the energy borders setting for strong, moderate and weak H-bonds, with 1.2–1.5 considered strong, >1.5–2.2 moderate and >2.2 weak. All intermolecular hydrogen bonds between baicalin and nsP3 in this study fell under the moderate bond group with one exception, interaction with the THR111 residue, which was categorised as a weak bond. Most of the hydrogen-bond donors came from the protein residues, in agreement with the study reported by Nguyen *et al.*^22^.

We also discovered one pi-pi interaction between baicalin and TYR114 of nsP3. In addition to hydrogen bonding and the pi-pi interaction, the van der Waal forces and electrostatic interaction energy were also documented. The van der Waal interaction is a weak intermolecular force between molecules that occurs when there is a fluctuation in the electron cloud of a nucleus that affects the transient dipole moment and electron cloud of nearby atoms. Despite its weak energy, large numbers of interaction can occur with the existence of good steric and electrostatic complementarity between an enzyme’s binding pocket and a ligand’s structure^30^. Electrostatic interaction occurring between a charged ligand and a receptor’s binding pocket helps to provide the thermodynamic driving forces involved in forming protein-ligand complexes.

The macro domain of CHIKV nsP3 contains the ADP-ribose binding site, which has been suggested to play an influential role in the metabolism of ADP-ribose 1″-phosphate and/or other ADP-ribose derivatives possessing regulatory activities in the cell^31^. In reference to the observed interacting residues in the ADP-ribose binding site, we found that all but one hydrogen bond (LEU108, THR111, TYR142) and the residue involved in the pi-pi interaction (TYR114) of baicalin with the nsP3 protein occurred within the active site of nsP3. Similarly, the hydrogen bonds formed when naringenin (THR111) and quercetagetin (CYS34, LEU108, ARG144) were docked to the active site of nsP3 were also observed when ADP-ribose was re-docked to the protein. Hence, it can be hypothesised that the binding of these three ligands will potentially result in a pharmacokinetic effect via their interactions with the ADP-ribose binding site.

Baicalin is a metabolite of baicalein, which can be extracted from the root of *Scutellaria baicalensis*, a Chinese medicinal herbal plant. Many pharmacological and *in vitro* antiviral activities of this flavonoid have been described. Other than having the ability to inhibit dengue virus replication and internalisation^11^, baicalin also serves as a potential antiviral agent against influenza viruses, where it acts as a neuraminidase inhibitor^32^. Another study in China presented a potent antiviral effect of this flavonoid against enterovirus 71 by inhibiting EV71/3D polymerase expression and the Fas/Fasl signalling pathways^33^.

In the medical industry, protein-ligand docking, rather than other docking types, has drawn special attention due to its fundamental role in structure-based drug design. Apart from being used to screen large databases to find a new antiviral lead compound, it is also useful for other purposes. A group of researchers studied the potential of cyclopentadeca-4, 1,2-dienone as an antidiabetic drug using Schrodinger software, with the result showing 52.3% α-amylase inhibitory activity^34^. Another docking study using AutoDock Vina was conducted to recognise the probable inhibitor against transketolase enzyme in *Plasmodium falciparum*, the protozoan responsible for malaria. The results of this study showed that 6′-methyl-thiamin diphosphate, an organic compound, gives the best pose with a binding affinity of −6.6 kcal/mol. Qiu *et al.*^35^ also documented a computational study to examine the molecular interaction between the active site of ginger and the human terminal oxidase enzyme cytochrome P450. The findings of this study led to the resolution of herbal medicine issues and safety concerns.

Molecular docking aims to predict the optimal ligand-receptor complex orientation and conformation in two steps, first by gathering conformation of ligands in the active site of the protein and then by ranking the conformations via a scoring function to predict binding tightness for each individual orientation^36^. Many commonly used molecular docking software programs are available, including AutoDock Vina, GOLD, FlexX, FRED and DOCK. However, AutoDock Vina was found to be the most useful software in blind docking pose prediction by consistently performing better than the other docking programs^37^. In addition, its accuracy and speed are approximately double that of its predecessor, AutoDock4^38^.

In conclusion, we found that among the three different ligands tested, baicalin exhibited the most potent potential antiviral activity against CHIKV nsP3 with a binding affinity of −9.8 kcal/mol, followed by quercetagetin and naringenin with respective affinities of −8.6 and −8.4 kcal/mol. Considering the involvement of CHIKV nsP3 in the intracellular CHIKV replication cycle, this result suggests that baicalin can potentially interfere with the post-entry stage(s) of CHIKV infection, which should prompt further investigations to reveal the mechanism of action of baicalin against CHIKV *in vitro* replication.

## Methods

### Receptor and Ligand Preparation

Molecular docking and virtual screening were conducted using AutoDock Vina. In this study, the protein was kept rigid while the ligands were fully flexible. The protein was prepared by retrieving the three-dimensional crystal structure of the nsP3 from the Protein Data Bank (PDB ID: 3GPG) and this was used as the receptor for molecular docking. The ligands, ADP-ribose, baicalin, quercetagetin and naringenin, were drawn using ChemDraw. The protein was subsequently cleaned by removing the water molecules, followed by management of its conformer and the minimisation process. The CHARMM27 force field was then applied in the Accelrys Discovery Studio 2.0 software package.

### Molecular Docking Using AutoDock Vina

The input files for AutoDock Vina were prepared using AutoDock Tools v. 1.5.6. After the minimising process, the protein was placed in a grid box measuring 26.85 Å × 28.17 Å × 24.53 Å along the *x*, *y* and *z* axes, respectively, using AutoDock Vina at 1.00 A to define the binding site. The configuration file used for the docking process was also prepared along with the addition of hydrogen bonds and the Gasteiger charge. ADP-ribose was first re-docked into the ADP-ribose binding site of nsP3^22^, and the resulting interactions were compared with those found in the subsequent focused dockings of baicalin, quercetagetin and naringenin into the similar active site using the same grid box. The docking procedure was performed using the instructed command prompts. The docking results included the binding energy value given in kcal/mol, the locations of hydrogen bonds, pi-pi interactions and closely interacting residues.

### Analysing and Output Visualisation using Discovery Studio

The docking poses were ranked according to their docking scores. The scoring function in AutoDock was used to predict the binding affinity of one ligand to the receptor molecule. The conformation with the lowest binding affinity was selected for further analysis after the docking process. Ki was calculated by the equation: Ki = exp [(ΔG*1000)/(R*T)], where ΔG is docking energy, R (gas constant) is 1.98719 cal K^−1^ mol^−1^ and T (temperature) is 298.15 K. Only the best pose (the one with the lowest binding energy) was considered for each ligand. The molecular visualisation of the docked complexes was performed using the Accelrys Discovery Studio software package^31^,^38^.

## Additional Information

**How to cite this article**: Seyedi, S. S. *et al.* Computational Approach Towards Exploring Potential Anti-Chikungunya Activity of Selected Flavonoids. *Sci. Rep.* **6**, 24027; doi: 10.1038/srep24027 (2016).

## Figures and Tables

**Table 1:** Close contacting residues of nsP3 when ADP-Ribose was re-docked to the protein.

Ligand	Close Contact(s)
ADP-Ribose	Ala22, Ala23, Asn24, Gly30, Asp31, Gly32, Val33, Cys34, Leu108, Thr111, Gly112, Val113, Tyr114, Tyr142, Cys143, Arg144

**Table 2:** Binding affinity and interaction energy of best docking pose against CHIKV NSP3.

Ligand	Ki value (μM)	Affinity (kcal/mol)	Interaction Energy (kcal/mol)	VDW Interaction Energy (kcal/mol)	Electrostatic Interaction Energy (kcal/mol)
ADP-ribose	–	−8.7	−967.552	−183.61	−783.942
Baicalin	0.064	−9.8	−552.405	105.187	−657.592
Naringenin	0.685	−8.4	−647.04	−24.713	−622.327
Quercetagetin	0.489	−8.6	−459.842	12.0442	−471.886

**Table 3:** Intermolecular H bond between each compound with CHIKV nsP3.

Compound	Interacting residue	Distance (Å)	H bond (D-H—A)
Baicalin	BACALIN:UNK1:H9 - 3GPG:LEU108:O	2.17575	H9-H–O
	BACALIN:UNK1:H18 - 3GPG:TYR142:O	1.95727	H18-H–O
	3GPG:LEU108:HN - BACALIN:UNK1:O32	1.81482	HN-H—O32
	3GPG:SER110:HN - BACALIN:UNK1:O18	1.8782	HN-H-O18
	3GPG:THR111:HN - BACALIN:UNK1:O19	2.00969	HN-H-O19
	3GPG:THR111:HG1 - BACALIN:UNK1:O19	2.42882	HG1-H—O19
Naringenin	3GPG:SER110:HN - N:UNK1:O20	2.33122	HN-H--O20
	3GPG:THR111:HN - N:UNK1:O19	2.24548	HN-H—O19
Quercetagetin	3GPG:CYS34:HN - Q:UNK1:O23	2.1684	HN-H—O23
	3GPG:LEU108:HN - Q:UNK1:O16	2.21037	HN-H—O16
	3GPG:ARG144:HN - Q:UNK1:O14	2.02438	HN –H—O14
	3GPG:ASP145:HN - Q:UNK1:O14	2.42109	HN –H—O14

**Table 4:** Pi-pi interaction between baicalin and CHIKV nsP3 residue, TYR114.

Ligand	Residue	Distance	Interaction
Baicalin	BACALIN:UNK1 - 3GPG:TYR114	5.3739	Pi-pi
