# Corrigendum: Computational Approach Towards Exploring Potential Anti-Chikungunya Activity of Selected Flavonoids

**DOI:** 10.1038/srep26368

**Published:** 2016-05-31

**Authors:** Seyedeh Somayeh Seyedi, Munirah Shukri, Pouya Hassandarvish, Adrian Oo, Esaki Muthu Shankar, Sazaly Abubakar, Keivan Zandi

Scientific Reports 6: Article number: 2402710.1038/srep24027; published online: 04132016; updated: 05312016

The original version of this Article contained an error in the spelling of the author Esaki Muthu Shankar, which was incorrectly given as Shankar Esaki Muthu. This has now been corrected in the PDF and HTML versions of the Article.

